# Worldwide distribution, symptoms and diagnosis of the coinfections between malaria and arboviral diseases: a systematic review

**DOI:** 10.1590/0074-02760240015

**Published:** 2024-06-24

**Authors:** Marcelo Cerilo-Filho, Marcelo de L Arouca, Estela dos S Medeiros, Myrela CS de Jesus, Marrara P Sampaio, Nathália F Reis, José RS Silva, Andréa RS Baptista, Luciane M Storti-Melo, Ricardo LD Machado

**Affiliations:** 1 Universidade Federal Fluminense, Centro de Investigação de Microrganismos, Instituto Biomédico, Departamento de Microbiologia e Parasitologia, Niterói, RJ, Brasil; 2 Universidade Federal Fluminense, Instituto Biomédico, Departamento de Microbiologia e Parasitologia, Programa de Pós-Graduação em Microbiologia e Parasitologia Aplicadas, Niterói, RJ, Brasil; 3 Universidade Federal Fluminense, Programa de Pós-Graduação em Ciências e Biotecnologia, Niterói, RJ, Brasil; 4 Universidade Federal de Sergipe, Centro de Ciências Biológicas e da Saúde, Departamento de Biologia, Programa de Pós-Graduação em Biologia Parasitária, São Cristóvão, SE, Brasil

**Keywords:** arbovirus infections, epidemiology, Plasmodium, vector borne diseases.

## Abstract

The coinfection between malaria (ML) and arboviral diseases represents a major global public health problem, particularly in tropical and subtropical countries. Despite its relevance, this topic is still insufficiently discussed in the current literature. Here, we aimed to investigate the worldwide distribution, symptoms, and diagnosis during coinfection between ML and arboviral diseases. We conducted a systematic review following the Preferred reporting items for systematic reviews and meta-analyses (PRISMA) statement and assessed the selection and eligibility criteria, created and diagrammed maps, and analysed major symptoms with 95% confidence intervals (CI) using prevalence ratio and effect size, also performing latent class analysis. A total of 85,485 studies were retrieved, of which 56 were included: 57.14% in Asia, 25% in Africa, 14.30% in South America, and 3.56% in Europe. A total of 746 individuals were reported to be coinfected with *Plasmodium* and arbovirus. Concurrent ML, Dengue (DEN), Chikungunya (CHIK), and Zika (ZIK) patients are more likely to present headache and skin rash. Regarding diagnosis, 58,253 were made, of which 38,176 were positive (ML and at least one arboviral disease). The magnitude of these pathogens’ coexistence points out the pressing need for improvements in public health policies towards diagnosis and prevention of both diseases, especially in endemic areas.

Arthropod-borne diseases (ABD) are among the major global health problems, responsible for more than 17% of all infectious diseases, and more than 700 thousand annual deaths in the world, with children being the most affected.^1^ Between them, Malaria (ML), Dengue (DEN), Chikungunya (CHIK), Zika (ZIK) and Yellow fever (YF) are among the most significant ABD.^1^ ^,^ ^2^ ^,^ ^3^

Malaria caused approximately 247 million cases and 619 thousand deaths worldwide in 2021.^4^ Among the species responsible for causing human ML, *Plasmodium falciparum* and *P. vivax* are responsible for the highest mortality and morbidity, respectively.^3^ ^,^ ^4^ Meanwhile, approximately 2,8 million cases of DEN, 274 thousand of CHIK, more than 40 thousand of ZIK, and 203 of YF were reported in 2022.^1^ ^,^ ^2^ ^,^ ^3^

These diseases share similar symptoms with each other and also with other infectious and non-infectious diseases.^1^ ^-^ ^8^ The most recurrent symptoms are febrile syndrome, myalgia, arthralgias, dizziness, vomiting, fatigue, anaemia, and headaches.^1^ ^,^ ^2^ ^,^ ^3^ ^,^ ^4^ ^,^ ^5^ This unspecific symptomatology could lead to misdiagnosis, especially during coinfection. Regardless of this limitation, the importance of these coinfections has been reported.^5^ ^,^ ^6^ ^,^ ^7^

Despite the clinical presentation similarities, clinical management during ML requires the use of antimalarial drugs, while no treatment is available for the viruses. Usually, the most effective method of controlling viruses is through vaccination. However, there are no vaccines or drugs available for CHIKV and ZIKV, and clinicians rely on supportive therapy, while for YFV a vaccine is recommended solely in the case of populations living in endemic areas.^1^ ^,^ ^6^ ^,^ ^7^ ^,^ ^8^ ^,^ ^9^ Furthermore, for DENV, two vaccines are available to the population: Dengvaxia (Sanofi Pasteur), since 2022,^10^ and QDENGA (Takeda), since 2023.^11^

A better understanding of the current knowledge about ML and arboviral diseases coinfection, encompassing their hotspots, diagnostic bottlenecks, and how it affects the patient’s follow-up and clinical management, depends on the careful examination over the sum of all information available on the reported cases. Thus, the aim of this systematic review is to assess the worldwide distribution of coinfections between ML and four arboviral diseases, as well as to report whether mono and coinfections present differences in symptoms as well as diagnostic/screening methods.

## MATERIALS AND METHODS

*Search strategy* - A systematic search was conducted following the Preferred reporting items for systematic reviews and meta-analyses (PRISMA) statement^12^ to identify relevant studies on the frequency, worldwide distribution, main symptoms, and diagnostic/screening methods on ML and arboviral diseases (DEN, CHIK, ZIK e YF) coinfection. The search for articles was performed in the PubMed, Google Scholar, Science Direct, and Scientific Electronic Library Online (SciELO) databases for studies published up to August 30th, 2023.

The research question was structured in the PICO format, where P = Patients with ML and arboviral diseases; I = *Plasmodium* spp. and arbovirus coinfection (DENV, CHIKV, ZIKV, YFV); C = Patients without coinfection; and O = Frequency, worldwide distribution, symptoms and diagnostic/screening methods of coinfection reported. Thus, the following questions were formulated: Is there a high frequency and worldwide distribution of coinfection between ML and the arboviral diseases addressed in this review? If yes, which symptoms are more prevalent in coinfection compared with monoinfection cases? Also, is the most frequently used method for screening/diagnosis the one that is recommended by the World Health Organisation (WHO)?

The following search terms were used: (“Malaria” AND “Arbovirus infections”); (“Malaria” AND “Arbovirus infections” AND “Symptomatology”); (“Malaria” AND “Arbovirus infections” AND “Diagnostic techniques and Procedures”); (“Malaria” AND “Arbovirus infections” AND “Quick diagnosis units”); (“Malaria” AND “Arbovirus infections” AND “Prevalence”); (“Malaria” AND “Dengue” AND “Prevalence”); (“Malaria” AND “Yellow fever” AND “Prevalence”); (“Malaria” AND “Chikungunya fever” AND “Prevalence”); (“Malaria” AND “Zika virus infection” AND “Prevalence”); (Malaria” AND “Arbovirus infections” AND “frequency”); (“Malaria” AND “Arbovirus infections” AND “Frequency”); (“Malaria” AND “Dengue” AND “ Frequency”); (“Malaria” AND “Yellow fever” AND “ Frequency”); (“Malaria” AND “Chikungunya fever” AND “ Frequency”).

*Selection and eligibility criteria* - The titles and abstracts of all returned studies were assessed for suitability. Studies were selected if they met the following criteria: (1) Peer-reviewed articles published in journals with a description of the sample strategy and study design; (2) Studies that included cases of coinfection between ML and arboviral diseases; (3) Surveys performed containing symptomatology, methods of screening/diagnosis and a description of the pathogen species; (4) Studies that included demographic information (children and/or adults, continent/country of residence/frequency); (5) Studies published up to August, 30th 2023. Full texts of potentially relevant studies were further analysed for coinfection prevalence data. Retrospective analysis and case reports with full text availability and reporting data about all the potential coinfections were included in the study.

The present work excluded studies carried out in non-humans, reviews, letters, opinion pieces, grey literature, as well as studies that did not have elucidated outcomes. A reference manager, EndNote Software (Version x9), was used to check and exclude duplicate articles. The risk of bias was assessed in each paper by two reviewers using three of the Joanna Briggs Institute’s (JBI) Criticals Appraisals Checklists for Case-Report and Analytical Cross Sectional Studies.^13^ Only papers considered to have a moderate score (≥ 50%) were included in this study.

*Data extraction* - The data extracted from the selected publications included: (i) Citation, (ii) Place/Continent where the study was carried out, (iii) Study design, (iv) Sample number, (v) Positive for coinfection, (vi) Age, (vii) Symptomatology, (viii) Diagnostic test, (ix) Remarks. All data were stored in Microsoft Excel^®^ 2020, and checked by three researchers.

*Frequency and global distribution mapping* - The frequencies and distributions of ML and arboviral diseases coinfections were summed, plotted on openly available maps (https://www.freeworldmaps.net), and then diagrammed using Ibis Paint X software (Version 10.0.2).

*Data analysis* - The frequency of each symptom for the diseases mentioned in the studies included in this review (ML, DEN, ZIK, CHIK and YF, as well as coinfections) was analysed through cross-reference tables, using Microsoft Excel software (2020) as a tool. This aimed to define the most frequently mentioned symptomatology among individuals.

Subsequently, the symptoms were analysed using the Chi-Square and Fisher’s Exact tests, aiming to determine if there is a distinct symptoms’ profile during coinfection. The prevalence ratio (PR), and the respective confidence interval (CI), were calculated for the occurrence of symptoms, based on the control group. Effect size (ES) measures were calculated using Cohen’s W statistic,^14^ ^,^ ^15^ which considers an effect to be insignificant for values less than 0.19, small effect for values between 0.20 and 0.49, medium effect for values between 0.50 and 0.79, large effect for values between 0.80 and 1.29, and very large effect for values equal to or greater than 1.30. All analyses were performed in R version 4.2.2, and the significance level adopted was 5%.^16^

Latent class analysis (LCA)^17^ ^,^ ^18^ ^,^ ^19^ was performed to understand the profile of symptoms. This statistical procedure seeks to group individuals according to similar patterns of responses, forming with greater homogeneity of interest and greater interclass heterogeneity. The choice of class number was made by means of the following statistical estimator: Akaike information criterion (AIC) and Bayesian information criterion (BIC). To test the association of latent classes with the different types of infection, a multinomial logistic regression (OR) model and their respective 95% CI were estimated as a measure of effect.

## RESULTS

**Fig. 1:**
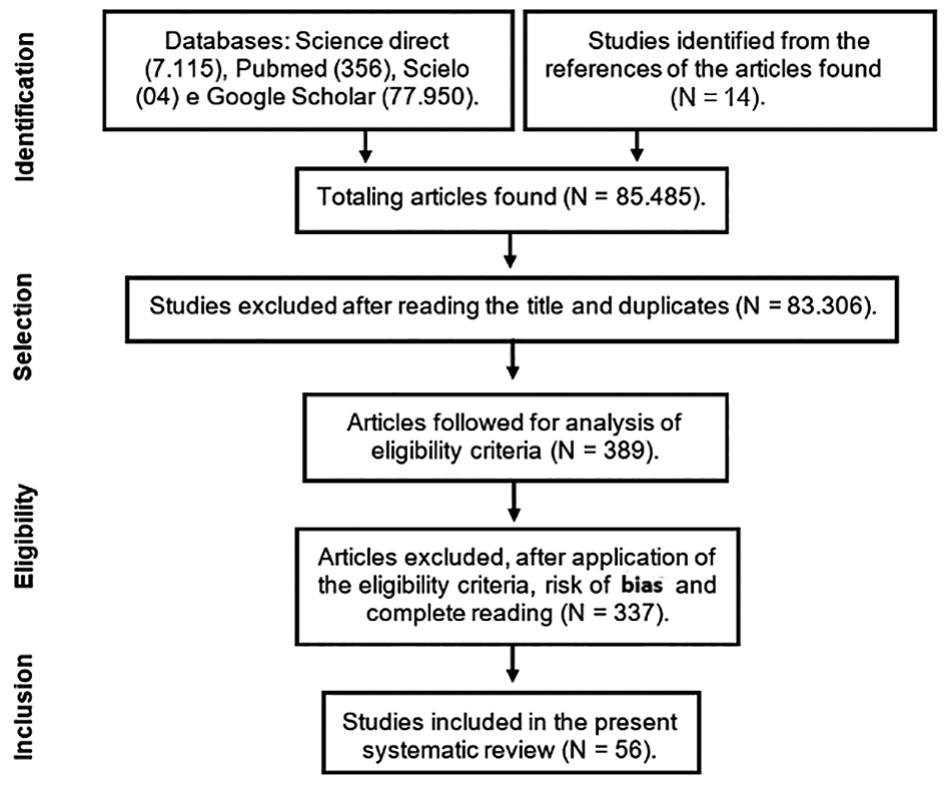
flowchart of the selection of studies for the systematic review on the worldwide distribution, symptomatology, and diagnosis of coinfections between malaria (ML) and arboviral diseases.

In the present systematic review, a total of 85,485 studies were identified, 97.45% of which were eliminated in the analysis of titles/abstracts along with duplicate articles, remaining 2,180 articles. These proceeded to the full reading stage, evaluation of selection, eligibility criteria, and risk of bias. With that, 56 articles were included in this study (87.66% were excluded; Fig. 1).

Among the 56 included studies, 67.85% were observational analytical cross-sectional studies while the remaining 32.15% were case reports, all published between the years of 2005-2020. A total of 52,913 individuals were analysed from the included articles, 746 of them parasitised by *Plasmodium* spp. and at least one Arbovirus (Table I). These coinfections were: 656 ML/DEN [Supplementary data (Table I)], 58 ML/CHIK [Supplementary data (Table II)], 25 ML/ZIK, and 07 ML/YF [Supplementary data (Table III)]. Coinfections were detected in all age groups (according to the eligible data from 51 articles). However, there was a higher prevalence in young adults aged 28-30 years.^20^ ^-^ ^76^

**TABLE I:** Studies for the systematic review on the worldwide distribution, symptomatology, and diagnosis of coinfections between malaria (ML) and arboviral diseases (ABV)

Num	Citation	Place/ Continent	Study design	N	Positive for coinfection	Coinfection (%)	Demography	Symptomatology	Diagnostic test ML/ABV	Remarks
1	^38^	Pakistan, Asia	Analytical cross-sectional studies	114	26	22.80%	Ages between 13 and 70 years	Fever ≤ 10 days duration, severe body aches, rash, and bleeding.	Blood smear, ELISA (IgM/IgG)	*Plasmodium vivax* and *P. falciparum*, DENV (no serotype)
2	^29^	India, Asia	Analytical cross-sectional studies	223	9	4.03%	Ages between 22 and 56 years	Acute febrile illness (< 2 weeks), associated with nausea, vomiting, and headache.	Blood smear, ELISA (IgM)	*Plasmodium. vivax* and *P. falciparum*, DENV (no serotype)
3	^30^	India, Asia	Case report	1	1	100%	42 years	Fever, chills, rigor, altered sensorium (Cerebral malaria and dengue).	Blood smear, RDT, ELISA (NS1/ IgM/ IgG)	*Plasmodium falciparum* and DENV (no serotype)
4	^35^	Yemen, Asia	Analytical cross-sectional studies	270	82	30.37%	Ages between 15 and 60years	Episodes of fever, headache, arthralgia, myalgia, and retro-orbital pain.	Blood Smear, RDT, ELISA (IgM/IgG)	*Plasmodium falciparum* and *P. vivax*, DENV (no serotype)
5	^31^	India, Asia	Case report	2	2	100%	35 and 63 years	Fever, chills, with vomiting and abdominal pain.	Blood smear, ELISA(NS1/ IgM/IgG)	*Plasmodium vivax* and DENV-2, DENV-3
6	^28^	Pakistan, Asia	Analytical cross-sectional studies	856	17	1.99%	Ages between 12 and 32 years	Fever of 2-10 days, myalgia, arthralgia, retro-orbital pain.	Blood smear, RT-PCR, ELISA(NS1/ IgM)	*Plasmodium vivax* and *P. falciparum* and DENV-2
7	^25^	Nigeria, Africa	Analytical cross-sectional studies	118	15	12.71%	All between 1 and 78 years	Fever > 37.5°C.	RDT, ELISA (IgM/IgG) Immunochromatography	*Plasmodium* sp*.* and ZIKV
8	^26^	Nigeria, Arica	Analytical cross-sectional studies	60	10	6%	Ages between 3 and 70 years	Fever, chills, headache, joint pain, muscle pain.	Blood smear, RDT, PCR, ELISA (NS1/ IgM/ IgG)	*Plasmodium vivax* and *P. falciparum*, DENV/ CHIKV (no serotype)
9	^53^	India, Asia	Case report	3	3	100%	Between 8 months and 12 years	Fever for 5-8 days, cough, and body ache.	Blood smear, RDT ELISA(NS1, IgM/IgG)	*Plasmodium vivax* and DENV (no serotype)
10	^57^	French Guiana, South America	Analytical cross-sectional studies	1723	17	0.99%	NM	Episodes of fever lasting up to 4 days.	Blood smear, ELISA(IgM), RT-PCR, virus isolation	*Plasmodium vivax*, *P. falciparum* and *P. malariae*, DENV-1, DENV-3
11	^74^	India, Asia	Case report	1	1	100%	28 years	Fever, chills in the last 7 days, abdominal pain, vomiting.	Blood smear, ELISA(IgM)	*Plasmodium falciparum* and DENV (no serotype)
12	^58^	France, Europe	Case report	1	1	100%	37 years	Fever, conjunctival jaundice, vomiting, diarrhoea.	Blood smear, IgM/IgG ELISA	*Plasmodium falciparum* and DENV-3
13	^42^	Malaysia, Asia	Case report	1	1	100%	59 years	Dyspnoea, chest discomfort, dry cough.	Blood smear, PCR, ELISA (NS1)	*Plasmodium knowlesi* and DENV (no serotype)
14	^24^	Malaysia, Asia	Case report	1	1	100%	59 years	Fever, headache, myalgia, arthralgia, and poor oral intake.	Blood smear, ELISA(NS1/ IgM)	*Plasmodium knowlesi* and DENV (no serotype)
15	^22^	Tanzania, Africa	Analytical cross-sectional studies	364	33	09.06%	Ages between 2 and 13 years	Fever, measured axillary or rectal temperature (37.5 or 38°C / 99.5 or 100.4°F).	Blood smear, ELISA(IgM/IgG), PCR	*Plasmodium* sp*.*, and DENV/ CHIKV (no serotype)
16	^43^	India, Asia.	Case report	1	1	100%	27 years	Myalgia (1 day before returning home to California from India after a 3 month period).	Blood smear, ELISA(IgM/ IgG)	*Plasmodium vivax* and DENV (no serotype)
17	^39^	French Guiana, South America	Analytical cross-sectional studies	208	104	50%	Ages between 15 and 75 years	Episodes of fever > 40°C, tachycardia, initial hypotension, nausea.	Blood smear, RT-PCR, ELISA(NS1/ IgM/IgA)	*Plasmodium vivax*, *P. falciparum*, and DENV-1, DENV- 2/ DENV-3
18	^40^	Bangladesh, Asia	Analytical cross-sectional studies	720	1	0.14%	4 years	Febrile patients > 38°C, headache, bodyaches, muscle pain.	RDT compatible with Blood smear, ELISA(IgM)	*Plasmodium vivax* and DENV (no serotype)
19	^46^	Peru, South American	Analytical cross-sectional studies	95	17	17.89%	Ages between 5 and 17 years	Fever, measured axillary > 37.5°C, abdominal pain, nausea, vomiting.	Blood Smear, PCR, ELISA (IgM/IgG), Immunofluorescence	*Plasmodium vivax*, *P. falciparum* and DENV-1, DENV-3
20	^32^	Nigeria, Africa	Analytical cross-sectional studies	340	2	0.59%	All ages	Febrile complaints (temperature > 37.5°C / 99.5°F).	Blood Smear, ELISA(IgM)	*Plasmodium* sp., and DENV (no serotype)
21	^23^	Thailand, Asia	Case report	1	1	100%	11 years	Fever, chills.	Blood smear, ELISA (NS1/ IgM/IgG)	*Plasmodium falciparum* and DENV (no serotype)
22	^55^	Cambodia, Asia	Analytical cross-sectional studies	9997	15	0.15%	Ages between 8 and 17 years	Fever in the last 24 hours and for < 10 days, muscle pain.	Blood Smear, ELISA (IgM/IgG), Nested PCR	*Plasmodium falciparum*, *P. vivax* and DENV-1, DENV-2, DENV-3, DENV-4
23	^46^	India, Asia	Case report	1	1	100%	26 years	Fever, headache, severe body pain and nausea for 10 days, chills every other day.	Blood smear, ELISA (IgM/IgG)	*Plasmodium vivax*, *P. falciparum* and DENV (no serotype)
24	^27^	Tanzania, Africa	Analytical cross-sectional studies	400	8	2.0%	Ages between 10 and 50 years	Fever > 38°C, headache, skin rashes, joint pain.	Blood smear, RDT, ELISA (IgM/ IgG)	*Plasmodium* sp*.* and CHIKV (no serotype)
25	^60^	Nigeria, Africa	Analytical cross-sectional studies	176	5	2.84%	Ages between 10 and 70 years	Febrile illness.	RDT, ELISA (IgM ELISA) RT-PCR	*Plasmodium* sp*.*, and DENV (no serotype)
26	^49^	Brazil, South America	Case report	1	1	100%	52 years	Chills, fever, headache, arthralgia, myalgia, choluria.	Blood smear, RDT, PCR,ELISA (IgM/ IgG/NS1)	*Plasmodium ovale* and DENV (no serotype)
27	^41^	Brazil, South America	Analytical cross-sectional studies	132	11	8.33%	Ages between 16 and 92 years	Febrile, chills, myalgias, arthralgias, headache.	Blood smear, RT-PCR, ELISA(NS1)	*Plasmodium vivax* and DENV-3, DENV-4
28	^37^	Brazil, South America	Analytical cross-sectional studies	1578	44	2.79%	Ages between 14 and 60 years	Episodes of fever in the past 10 days.	Blood smear, RT-PCR, ELISA (IgM/NS1)	*Plasmodium vivax* and DENV-2, DENV-4
29	^50^	Thailand (Burmese border), Asia	Analytical cross-sectional studies	203	1	0.49%	Ages between 15 and 41 years	Febrile episodes up to 3 days (aural temperature 37.5ºC), headache, anorexia, muscle pain.	Blood smear, ELISA (IgM/ NS1)	*Plasmodium falciparum*, *P. vivax* and DENV (no serotype)
30	^36^	Brazil, South America	Analytical cross-sectional studies	72	30	41.67%	Ages between 20 and 44 years	Acute febrile syndrome.	Blood smear, PCR/ RT-PCR, ELISA (IgM/ NS1)	*Plasmodium vivax*, *P. falciparum* and DENV-1, DENV-2, DENV-3, DENV-4
31	^44^	India, Asia	Analytical cross-sectional studies	2547	11	0.43%	≥ 18 years	Febrile illness with duration of 5-14 days, rash, hepatomegaly and abdominal pain.	Blood smear, RDT, ELISA (NS1/ IgM)	*Plasmodium* sp*.*, and DENV (no serotype)
32	^61^	India, Asia	Analytical cross-sectional studies	469	27	5.76%	NM	Fever for < 7 days, running nose, myalgia, headache, and bleeding manifestations.	Blood smear, ELISA (IgM/ NS1)	*Plasmodium falciparum*, *P. vivax* and DENV (no serotype)
33	^62^	India, Asia	Analytical cross-sectional studies	1564	78	4.98%	≥ 5 years	Fever temperature ≥ 38°C (100.4°F) and febrile illness with duration of 2-14 days.	Blood smear, ELISA (IgM/IgG/NS1), and Blood cultures.	*Plasmodium falciparum* and DENV/CHIK (no serotype)
34	^63^	Cambodia, Asian	Analytical cross-sectional studies	1193	27	2.26%	Ages between 7 and 49 years	Febrile illness (> 38ºC), sore throat, cough, and running nose.	RDT, Nested-PCR, and RT-PCR.	*Plasmodium vivax*, *P. falciparum* and DENV (no serotype)
35	^64^	Mozambique Africa	Analytical cross-sectional studies	163	2	1.23%	Ages between 5 and > 40 years	Acute febrile illness > 37.5°C, headache, arthralgia, myalgia.	RDT, ELISA (IgM/ IgG/NS1), qRT-PCR	*Plasmodium falciparum* and CHIKV (no serotype)
36	^54^	India, Asia	Case report	1	1	100%	25 years	Fever, chills, myalgias, headache, severe headache, and high fever of 102°F (38.9°C).	Malaria Ag (pLDH/ HRP2), Blood smear, ELISA (IgM)	*Plasmodium vivax*, *P. falciparum* and DENV (no serotype)
37	^76^	Tanzania, Africa	Analytical cross-sectional studies	448	13	2.90%	All ages between 2 and 70 years	Participants without complaints, randomly selected.	Blood Smear, ELISA (IgM)	*Plasmodium* sp*.* and CHIKV (no serotype)
38	^66^	Cameroon, African	Analytical cross-sectional studies	349	68	19.48%	Ages between 6 months to 15 years	Children presenting episodes of fever (37.8-41°C, or, 100.04-105.8°F), vomiting, diarrhoea, and fatigue.	Blood Smear, ELISA (NS1/ IgM/IgG)	*Plasmodium falciparum*, *P. vivax* and DENV (no serotype)
39	^67^	Nigeria, Africa	Analytical cross-sectional studies	529	35	6.62%	Ages between < 18 and 58 years	Episodes of fever (axillary temperature > 37.8°C/100.04°F).	Blood smear, ELISA (IgM/ IgG/ NS1)	*Plasmodium falciparum* and DENV (no serotype)
40	^56^	Nigerian, Africa	Analytical cross-sectional studies	188	19	10.11%	Ages between 4 and 82 years	Acute fever (> 38ºC)	NM ELISA(IgG/IgM,NS1)	*Plasmodium* sp., and DENV (no serotype)
41	^20^	India, Asia	Case report	1	1	100%	25 years	Fever (temperature = 101°F, or, 38.3°C), dyspnoea, erythematous rash.	Blood smear, ELISA(NS1/ IgG/ IgM)	*Plasmodium vivax*, *P. falciparum* and DENV (no serotype)
42	^75^	India, Asia	Analytical cross-sectional studies	100	3	3.0%	Ages between 5 and ≥ 60 years	Fever, abdominal pain and bleeding.	Blood smear, RDT/ ELISA	*Plasmodium* sp., and DENV (no serotype)
43	^69^	India, Asia	Analytical cross-sectional studies	1980	22	1.11%	Ages between 5 and > 15 years	Febrile illness (38.3-39.4°C / 100.94-102.92°F), headache, retro-orbital pain, fever for 2-15 days.	Blood smear, RDT, ELISA(IgM/ IgG, NS1) RT-PCR	*Plasmodium vivax*, *P. falciparum* and DENV (no serotype)
44	^47^	India, Asia	Case report	1	1	100%	17 years	Fever, chills in the last 5-6 days, abdominal pain, and vomiting.	RMAT, PCR, ELISA(IgM)	*Plasmodium vivax*, *P. falciparum* and DENV (no serotype)
45	^48^	Brazil, South America	Analytical cross-sectional studies	111	2	1.80%	Ages > 18 years	Episodes of fever, headache, and shivering.	Blood smear, RT-PCR, Nested-PCR	*Plasmodium vivax*, *P. falciparum* and DENV-1
46	^33^	Madagascar, Africa	Analytical cross-sectional studies	1216	2	0.16%	Pregnant women, all ages	NM	ELISA (IgG/IgM), IIFA, PCR	*Plasmodium falciparum* and ZIKV
47	^59^	Spain, Europe	Case report	1	1	100%	27 years	Fever, constipation, and joint pain.	Blood smear, RT-PCR, ELISA (IgM/ IgG/NS1)	*Plasmodium falciparum* and DENV-4
48	^70^	India, Asia	Analytical cross-sectional studies	8364	27	0.32%	NM	Fever compatible with malaria and/or dengue.	Blood smear, ELISA (NS1/ IgM)	*Plasmodium falciparum* and DENV (no serotype)
49	^71^	India, Asia	Analytical cross-sectional studies	1141	9	0.79%	Ages between 12 and 80 years	Acute febrile illness.	Blood smear, ELISA (IgM, NS1)	*Plasmodium* sp., and DENV (no serotype)
50	^6^	Senegal, Africa	Analytical cross-sectional studies	13845	19	0.13	Ages between 1 and 90 years	Acute febrile illnesses (> 38 °C), headache, myalgia, eye pain, arthralgia.	Blood smear, RDT, ELISA (IgM), RT-PCR	*Plasmodium* sp., and DENV/CHIKV/ZIKV/YFV (no serotype)
51	^52^	Ghana, Africa	Analytical cross-sectional studies	218	7	3.21%	Ages between 2 and 14 years	Febrile illness, headache, nausea, chills.	RDT, ELISA (IgM/ IgG), RT-PCR	*Plasmodium* sp., and DENV (no serotype)
52	^72^	Bangladesh, Asia	Analytical cross-sectional studies	659	5	0.76%	Ages between 0 and 90 years	Fever > 37.5°C, fatigue, fever, dizziness and headache.	RDT, PCR, Blood smear, ELISA (IgM)	*Plasmodium falciparum*, *P. vivax*, *P. malariae* and DENV (no serotype)
53	^73^	India, Asia	Case report	1	1	100%	22 years	Fever > 39° C, chills, rigors, cough up to 3 days.	Blood smear, ELISA(IgM)	*Plasmodium vivax* and DENV (no serotype)
54	^21^	East Timor, Asia	Case report	1	1	100%	7 years	Fever, headache, fatigue, anorexia.	Blood Smear, RDT, ELISA (IgM)	*Plasmodium falciparum* and DENV (no serotype)
55	^34^	Pakistan, Asia	Analytical cross-sectional studies	159	5	3.74%	Ages > 12 years	Acute febrile illness and found to have thrombocytopenia.	Blood smear, IgM	*Plasmodium falciparum* and DENV (no serotype)
56	^51^	Indonesia, Asia	Case report	1	1	100%	49 years	Fever, chills, rigors, myalgia.	Blood smear, ELISA (IgM/ NS1)	*Plasmodium falciparum* and DENV (no serotype)

ELISA: enzyme-linked immunosorbent assay; PCR: polymerase chain reaction; qRT-PCR: reverse transcription real-time PCR; RDT: rapid diagnostic test; CHIK: Chikungunya; DENV: Dengue virus; YFV: Yellow fever virus; ZIKV: Zika virus.

As for the *Plasmodium* species, there was a predominance of *P. vivax* ^26^ ^,^ ^28^ ^-^ ^29^ ^,^ ^31^ ^,^ ^36^ ^-^ ^38^ ^-^ ^41^ ^,^ ^43^ ^,^ ^45^ ^-^ ^46^ ^,^ ^50^ ^,^ ^53^ ^-^ ^54^ ^,^ ^61^ ^-^ ^62^ ^,^ ^64^ ^,^ ^70^ ^,^ ^73^ with 3,483 monoinfected individuals, followed by *P. falciparum* with 1,557 cases, and the concomitant infection between *P. falciparum* and *P. vivax* with 175 cases. As for DEN, 124 individuals were positive for DENV-1,^36^ ^,^ ^39^ ^,^ ^45^ ^,^ ^48^ ^,^ ^56^ ^,^ ^58^ 221 for DENV-2,^28^ ^,^ ^31^ ^,^ ^36^ ^-^ ^37^ ^,^ ^39^ ^,^ ^41^ ^,^ ^48^ ^,^ ^56^ 611 for DENV-3,^36^ ^,^ ^38^ ^,^ ^41^ ^,^ ^45^ ^,^ ^56^ ^,^ ^58^ and 368 for DENV-4.^36^ ^-^ ^37^ ^,^ ^39^ ^,^ ^41^ ^,^ ^55^ ^-^ ^56^ Among the articles, three^21^ ^,^ ^35^ ^,^ ^45^ reported one death, each from coinfection between ML and arboviral diseases. No *P. malariae*, *P. simium*, *P. cynolmolgi*, *P. inui* and arbovirus Oropouche and Mayaro coinfection reports were retrieved.

**Fig. 2:**
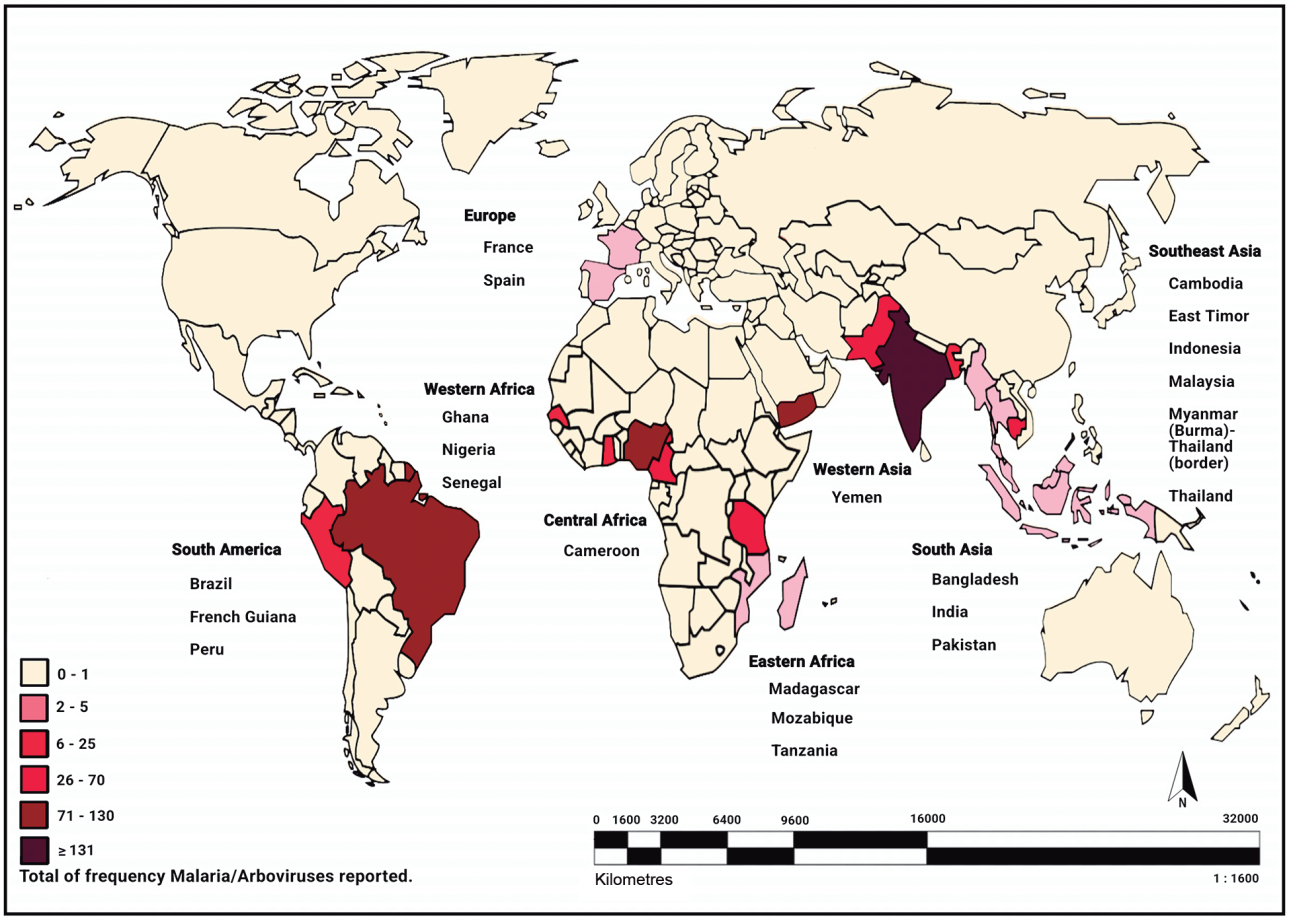
worldwide frequency and distribution map of malaria (ML) and arboviruses coinfections, according to the studies in this systematic review.

**Fig. 3:**
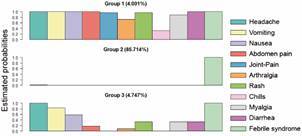
latent class analysis (LCA). The choice of class number was made using the statistics Akaike information criterion (AIC) and Bayesian information criterion (BIC), according to the studies in this systematic review.

As for the worldwide frequency of coinfection between ML and arboviral diseases, fewer cases have been reported in Europe, Southeast Asia, and Eastern Africa (between 02 to 25 cases). However, in South America, Western and Central, the incidence of coinfection notifications increases, reaching over 130 cases. In relation to continental distribution, 57.14% were conducted in Asia, followed by 25% in Africa, 14.30% in South America, and 3.56% in Europe. No investigations answering the research questions were found in Oceania, North or Central America [Fig. 2 and Supplementary data (Figs 1,2,3,4)].

Among the 56 articles used to analyse the symptoms, 3.57%^40^ ^,^ ^49^ were left out because they did not present symptoms as an inclusion criterion. Of the articles analysed, a total of 49,048 individuals participated in these studies and presented the symptoms for inclusion in our research.

A similar series of symptoms was observed in patients who had ML or an arboviral disease in single infections, as in those who were simultaneously monoinfected with any of the five pathogens (ML, DENV, CHIKV, ZIKV, YFV). The symptoms most commonly reported by coinfected individuals were fever, headache, vomiting, tinnitus, abdominal pain, bleeding, and diarrhoea.

Table II describes the patients who had simultaneous infections for ML and arboviral diseases with a prevalence ratio and effect size between medium for the following symptom: Rash (PR: -, *p-value*: 0.000, ES: 0.506). The other selected symptoms showed non-significant (*e.g.*: Febrile syndrome (PR: 1, *p-value*: 0.000, ES: 0.022) or small results (*e.g.*: Nausea (PR: 10.51, *p-value*: 0.000, ES: 0.317).

**TABLE II:** Prevalence ratio (PR) and effect size (ES) of symptoms that *Plasmodium*/arboviral diseases coinfected individuals may develop

Symptoms	N (%)	PR	*p-value*	ES
Headache				
Malaria Arbovirus Coinfection Control	2147 (17.6) 3615 (46.9) 413 (56.2) 1323 (4.5)	3.88 (3.63, 4.14) 10.32 (9.74, 10.94) 12.37 (11.39,13.44) 1	0.000	0.440
Vomiting				
Malaria Arbovirus Coinfection Control	1934 (15.9) 2690 (34.9) 413 (56.2) 970 (3.3)	4.76 (4.42, 5.13) 10.48 (9.78, 11.22) 16.87 (15.44, 18.44) 1	0.000	0.385
Nausea				
Malaria Arbovirus Coinfection Control	1620 (13.3) 2215 (28.7) 255 (34.7) 962 (3.3)	4.02 (3.72, 4.34) 8.7 (8.1, 9.34) 10.51 (9.35, 11.81) 1	0.000	0.317
Abdomen pain				
Malaria Arbovirus Coinfection Control	1399 (11.5) 1702 (22.1) 290 (39.5) 8 (0)	417.58 (208.49,836.39) 803.71 (401.46, 1608.99) 1436.68 (714.42, 2889.14) 1	0.000	0.364
Joint pain				
Malaria Arbovirus Coinfection Control	881 (7.2) 1610 (20.9) 79 (10.7) 0 (0)	NA NA NA 1	0.000	0.336
Arthralgia				
Malaria Arbovirus Coinfection Control	533 (4.4) 1293 (16.8) 128 (17.4) 361 (1.2)	3.53 (3.09, 4.02) 13.53 (12.07, 15.16) 14.05 (11.65, 16.96) 1	0.000	0.269
Rash				
Malaria Arbovirus Coinfection Control	791 (6.5) 2974 (38.6) 183 (24.9) 0 (0)	NA NA NA 1	0.000	0.506
Chills				
Malaria Arbovirus Coinfection Control	381 (3.1) 322 (4.2) 158 (21.5) 0 (0)	NA NA NA 1	0.000	0.229
Myalgia				
Malaria Arbovirus Coinfection Control	687 (5.6) 1507 (19.5) 212 (28.8) 1323 (4.5)	1.24 (1.13, 1.36) 4.3 (4.01, 4.61) 6.35 (5.6, 7.2) 1	0.000	0.225
Diarrhea				
Malaria Arbovirus Coifection Control	1642 (13.5) 2117 (27.5) 290 (39.5) 0 (0)	NA NA NA 1	0.000	0.397
Febrile syndrome				
Malaria Arbovirus Coinfection Control	12199 (100) 7711 (100) 735 (100) 29096 (99.9)	1 (1, 1) 1 (1, 1) 1 (1, 1) 1	0.000	0.022

NA: not applicable.

In order to describe the clinical profiles, the LCA was performed (Fig. 3), gathering symptoms into three groups. In this analysis, the second group contained 85.71% of all individuals in the study. Thus, in both mono and coinfection cases, a predominance of febrile symptoms was observed. During the analysis of the association of latent classes with the different diseases (Table III), it was observed that the individual who was coinfected with ML/arboviral diseases had a 12.49x chance of developing the symptoms present in Group 3 (Fig. 3).

**TABLE III:** Multinomial logistic regression, with the odds ratio (OR) as the measure of effect, and their respective 95% confidence intervals (CI), regarding patient-reported symptoms in this systematic review

Variables	Group 1		Group 3	
	OR	CI 95%	OR	CI 95%
Malaria	14.95	55.64 - 85.53	2.14	1.97 - 2.33
Arbovirus	16.32	54.27 - 86.91	6.01	5.54 - 6.53
Coinfection	16.84	53.75 - 87.42	14.92	12.49 - 17.82
Control	1		1	

Regarding diagnosis, 58,253 of them were performed by distinct methodologies. Of these, a total of 38,176 were positive (ML and/or some arboviral disease), 609 individuals were coinfected, with seven (n = 7) of them presenting simultaneous infection for ML/DEN/CHIK.^22^ ^,^ ^26^ ^,^ ^63^ The thick blood film was the main *Plasmodium* spp. diagnostic method used (n = 11,960), although molecular techniques were also observed. Overall, for DENV, the enzyme-linked immunosorbent assay (ELISA) for M immunoglobulin (ELISA IgM) and the ELISA for NS1 antigen (ELISA NS1) were more frequently chosen (5,894 and 4,516 tests, respectively). For CHIKV, the ELISA IgM (n = 238) and ELISA IgG (n = 208) were also used. The combined ELISA IgM/IgG test (n = 48) was the main choice for ZIKV antibody detection. Finally, for YFV the ELISA IgM test was more frequently applied (n = 11) (Table IV). The reverse transcription-polymerase chain reaction (RT-PCR) diagnosis was used in few studies.

**TABLE IV:** Types of diagnoses and frequency used to identify malaria (ML)/arboviral disease (ABV) of the studies in this systematic review

Groups	Types of diagnostic tests / frequency (%)																										
	BS		RDT		PCR		RT-PCR		Nested PCR		ELISA (NS1)		ELISA (IGM)		ELISA (IGG)		ELISA (IGM/IGG)		Blood culture		Virus isolation		NT		TOTAL		
	RP	%	RP	%	RP	%	RP	%	RP	%	RP	%	RP	%	RP	%	RP	%	RP	%	RP	%	RP	%	P (n)	TPCa	%
ML	11.960	89.43	9.499	71.03	905	7,57	NA	NA	676	5.05	NA	NA	NA	NA	NA	NA	NA	NA	NA	NA	NA	NA	44	0.33	13,374	22,989	171.89
ML/ABV	698	93.57	431	57.77	87	11.66	NA	NA	46	6.17	335	44.91	645	86.46	166	22.25	220	29.49	17	2.28	9	1.21	120	16.09	746	3,129	419.44
ABV	NA	NA	1.033	13.87	262	3.52	1,206	16.19	848	11.38	4,516	60.63	6.166	82.78	2.346	31.49	1536	20.62	68	0.91	220	2.95	115	1.54	7449	18,316	245.89
DENV	NA	NA	979	14	227	3.26	1,206	17.34	845	12.15	4,516	64.91	5.894	84.72	2.122	30.50	1373	19.74	68	0.98	220	3.16	115	1.65	6,957	17,565	252.48
CHIKV	NA	NA	10	2.37	33	7.82	NA	NA	3	0.71	NA	NA	238	56.40	208	49.29	115	27.25	NA	NA	NA	NA	-	-	422	607	143.84
ZIKV	NA	NA	44	77.19	NA	NA	NA	NA	NA	NA	NA	NA	23	40.35	16	28.07	48	84.21	NA	NA	NA	NA	-	-	57	131	229.82
YFV	NA	NA	NA	NA	2	15.38	NA	NA	NA	NA	NA	NA	11	84.62	NA	NA	NA	NA	NA	NA	NA	NA	-	-	13	13	100.00

BS: blood smear; RDT: rapid diagnostic tests; PCR: polymerase chain reaction; RT-PCR: reverse-transcription polymerase chain reaction; nested PCR: nested polymerase chain reaction; ELISA (NS1): enzyme-linked immunosorbent assay for NS1 antigen; ELISA (IgM): enzyme-linked immunosorbent assay for M immunoglobulin; ELISA (IgG): enzyme-linked immunosorbent assay for G immunoglobulin; ELISA (IgM/IgG): enzyme-linked immunosorbent assay for M and G immunoglobulins; NT: analysis type not mentioned; RP: reported positives; P (n): number of individuals with positive results; TPCa: total positive case analysis; DENV: Dengue virus; CHIKV: Chikungunya virus; ZIKV: Zika virus; YFV: Yellow fever virus; NA: not applicable.

## DISCUSSION

Diseases transmitted by vectors such as ML, DEN, CHIK, ZIK, and YF are of global epidemiological importance due to their mode of transmission, which involves a vector mosquito, humans, and the environment, characterising a one health problem.^1^ ^,^ ^2^ ^,^ ^3^ ^,^ ^4^ ^,^ ^5^ In turn, vector species involved in the transmission of ML parasites and arboviruses have different habits and environmental conditions in the development of their biological cycle.^1^ ^,^ ^5^ ^,^ ^7^ Therefore, it should be added that transmission may be related to the individual’s place of residence, as well as their work activities, such as mining, deforestation, hunting, and fishing.^29^ ^,^ ^30^ ^,^ ^31^

As expected, our results demonstrate that the majority of studies on these diseases come from countries located in tropical and subtropical regions of Asia, Africa, and the Americas, respectively. In these continents, hot and rainy weather form water collections, unplanned urban development, poor sanitation, rampant deforestation, and intense population flow between endemic and non-endemic regions of these pathologies.^6^ ^,^ ^20^ In such a scenario, the adaptation of the microorganism to the vector and environmental conditions favours the proliferation of *Anopheles* spp. and *Aedes* spp., as well as the development of these diseases, also as coinfections.^22^ ^,^ ^31^ ^,^ ^71^ ^-^ ^85^

ML, DEN, CHIK, ZIK, and YF are diseases that were first reported in Africa, representing the second continent with the highest number of reported coinfection cases in this review. This is likely due to the fact that Asia presents an overpopulation, leading to more cases. However, there may be underreporting in the African continent, as a significant portion of its population lives in war-torn regions, which also contributes to difficulties in healthcare access.^25^ ^,^ ^33^ ^,^ ^73^ ^,^ ^74^

America, the third continent with the highest number of coinfections, presents similar numbers of mono and coinfections compared to Africa. Presumably, the aforementioned information is somehow interconnected, since their vulnerable population faces environmental and socioeconomic challenges, leading to an increase in infectious disease burden.^25^ ^-^ ^33^ In this review, only two European studies described coinfection cases, which happened during trips to Central America and to Africa. The cold climate hinders the spread of vector mosquitoes along with the advantageous socio-economic conditions and effective prophylactic measures, compared to those observed in the African, Asian, and American continents. These are responsible for the lower frequency of coinfections.^58^ ^-^ ^61^

Coinfection between ML and DEN was the most common, followed by ML+CHIK, ML+ZIK, and ML+YF. These data reflect the pathogens’ ability to complete their biological cycle in the vector and the vectorial competence to transmit these arboviral diseases, with a better shape in the host-parasite relationship for concurrent infection between ML+DEN.^28^ ^,^ ^29^ ^,^ ^30^ ^,^ ^31^ This scenario is likely to change in view of the promising DENV vaccines, which are already in the testing/implementation phase.^10^ ^,^ ^11^ ^,^ ^86^ ^-^ ^88^ In contrast, the detection of seven cases of coinfection between ML and YF, the only arboviral disease that currently has an effective vaccine, reflects the global vaccination policy.^28^ ^-^ ^34^

In Brazil, South America, the main vectors of ML and DEN, *Anopheles darlingi* and *Aedes aegypti*, respectively, have different behaviours, the former more associated with rural areas and the latter with urban areas. However, nowadays, it has been observed that *A. darlingi* due to anthropological actions, such as the construction of hydroelectric plants, illegal mining, unlawful deforestation, and urbanisation, has changed its behaviour, causing urban ML, especially in the Brazilian Amazon. In this way, these two vectors end up coexisting in the same space, which can increase the risk of infection, especially by *Plasmodium* spp. + DENV, which accounts for the majority of coinfection cases. This fact raises new challenges for public health in the control of both diseases, since, in this country, the surveillance of ML and DEN is usually developed separately.^37^ ^,^ ^38^ ^,^ ^42^ ^,^ ^48^ ^,^ ^49^

It is important to note that, in this systematic review, coinfection was observed between ML and various arboviral diseases (DEN, CHIK, ZIK, and YF). However, no reports were presented regarding the detection of cases by *Plasmodium* spp. and the Mayaro and Oropouche viruses. This fact is likely due to the absence of routine laboratory diagnosis of these two arboviruses in the studied areas.^22^ ^-^ ^26^ We emphasise the need for the investigation of arboviruses, specifically the Mayaro and Oropouche viruses, to be implemented in routine diagnosis, given reports of their co-circulation in ML-endemic regions.^85^ ^,^ ^86^

*Symptomatology: aspects that lead to similarity in the clinical manifestation of mono and coinfections between ML and arboviral diseases* - *Plasmodium* spp. and the arbovirus investigated here are challenged to thrive in spite of the immunological factors involved in the host-parasite interaction, which are intrinsically permeated by the human host genetic variability.^32^ ^-^ ^80^ In spite of all this variety and its consequent possible outcomes, intriguingly, we could not observe major symptoms and/or clinical manifestations to be named as pathognomonic in patients with concurrent ML and arboviral diseases. As a matter of fact, in such infectious diseases, the pro-inflammatory cytokine cascades, during coinfections, play a crucial role in the severity of symptoms.^24^ ^,^ ^25^ ^,^ ^26^ ^,^ ^27^ Likewise, undifferentiated febrile syndrome, neurological symptoms, joint pain, and anaemia were constantly recorded for the majority of all cases.

Nevertheless, this systematic review brings to light symptoms which can be considered of attention to healthcare providers working in endemic areas for ABD. The first of these was that concurrent ML, DEN, CHIK, and ZIK patients are more susceptible to presenting headache and skin rash. Therefore, an important public health measure could be to implement *Plasmodium* spp. investigation whenever skin rash and headache appear as symptoms in an arbovirus-infected patient, at least in the endemic area of both diseases, when these diseases coincide spatially and affect the same population groups. Secondly, when the three clinical aspects are assessed (febrile syndrome, bleeding, and thrombocytopenia), the probability of concurrent ML and DEN is observed.^50^ ^-^ ^60^ Undoubtedly, this triad can worsen the clinical condition, especially in immunocompromised individuals, pregnant women, and children.^55^ ^-^ ^60^

Anaemia reported in individuals infected with *Plasmodium* spp. has multifactorial origins. One of the studied factors may be related to coinfection with arboviruses, especially DEN.^20^ ^-^ ^24^ ^,^ ^37^ ^-^ ^40^ Haemorrhages associated with febrile syndrome can worsen the clinical picture, making therapeutic approaches by healthcare professionals challenging. Added to this concern, severe DEN associated with simultaneous ML infection becomes a problem, as some of the antimalarial drugs cannot be prescribed to pregnant women, neonates, and glucose-6-phosphate dehydrogenase deficiency carriers.^32^ ^,^ ^33^ ^,^ ^34^

*Diagnosing coinfection between ML and arboviral diseases: a lasting issue* - Delaying the identification of the cause of the illness in endemic areas of *Plasmodium* and arbovirus can lead to clinical management uncertainty or even critical misconduct. One of its complex consequences is returning the patient to its community in the condition of a constant source of transmission.^38^ ^-^ ^50^ Therefore, there is a need for the implementation of diagnostic strategies for these microorganisms, their vectors, and also their environmental conditions to develop effective prevention and control measures, as recommended by the WHO and Pan American Health Organisation (PAHO).^89^ ^,^ ^90^

Thick blood film is the gold standard of the ML diagnosis, as verified. However, this technique has limitations, as it can result in false negatives, especially in cases of low parasitaemia and mixed infections. Additionally, it requires the expertise of a microscopist to identify the species, its stage, as well as atypical forms of *Plasmodium* spp.^83^ The rapid diagnostic test (RDT) is the second laboratory protocol option for ML detection found in this review, being the most widely used test in remote areas.^62^ ^-^ ^66^ ^,^ ^81^ However, it has limitations such as cost and inability to quantify parasitaemia and demonstrate the parasitic stage.^36^ ^,^ ^40^ It is worth noting that polymorphisms affecting the expression of the rapid test recognition parasite protein have been observed, leading to false negative results. Diagnostic serology is not efficient for ML, and molecular biology techniques still have high operational costs, limiting their use in routine diagnosis.^60^ ^,^ ^61^ ^,^ ^62^ ^,^ ^63^ ^,^ ^72^

This systematic review showed that the IgM/IgG ELISA was the most commonly used diagnostic protocol for detecting arboviruses. However, these serological tests do not define current infection but only indicate their circulation in endemic regions.^50^ ^,^ ^51^ ^,^ ^52^ ^,^ ^53^ Most articles describe that arbovirus investigation typically follows *Plasmodium* spp. Investigation.^60^ ^-^ ^65^ The DENV NS1 antigen detection test by ELISA was also used, as well as the RT-PCR for all other arboviruses.^62^ ^-^ ^67^ However, these protocols were not observed in the majority of studies. The limitations of serological tests in detecting antibodies against arboviruses should be considered, as seroconversion takes an average of 6-10 days.^76^ ^-^ ^80^ ^,^ ^84^ ^,^ ^85^

*In conclusion* - Coinfection and co-circulation between *Plasmodium* spp. and arbovirus are predominantly found in tropical and subtropical countries, where socio-environmental-sanitary conditions favour transmission. The review of vaccination programmes against YF is crucial in controlling this arbovirus. Protocols related to symptoms and diagnosis need to be redefined to distinguish coinfection from co-circulation, requiring molecular tests. Therefore, the current scenario of coinfection between ML and arboviral diseases still needs more extensive study, calling for efficient public health policies and investment in health education. The ultimate goal is to mitigate these diseases and improve the quality of life for the population.
