# Supplementary material for: Worldwide distribution, symptoms and diagnosis of the coinfections between malaria and arboviral diseases: a systematic review
Source: Mem Inst Oswaldo Cruz. 2024 Jun 24;119:e240015. doi: 10.1590/0074-02760240015 (PMC11197440; doi:10.1590/0074-02760240015)
Supplement: Supplementary file 1 [file 1678-8060-mioc-119-e240015-s.pdf]

TABLE I

Studies for the systematic review on the worldwide distribution, symptomatology, and diagnosis of coinfections between *Plasmodium* and Dengue virus (DENV)

Num.	Citation	Place/Continent	Study design	N	Positive for coinfection	Coinfection (%)	Demography	Symptomatology	Diagnostic test ML/ DENV	Remarks
1	[6]	Senegal, Africa	Analytical cross-sectional studies	13,845	1	0.007	Ages between 1 and 90 years	Acute febrile illnesses (> 38 °C), headache, myalgia, eye pain, arthralgia.	Blood smear, RDT, ELISA (IgM), RT-PCR	*Plasmodium* sp., and DENV (no serotype)
2	[20]	India, Asian	Case report	1	1	100	25 years	Fever (101°F or 38.3°C), dyspnoea, erythematous rash.	Blood smear, ELISA (NS1/IgG/ IgM)	*Plasmodium vivax*, *P. falciparum* and DENV (no serotype)
3	[21]	East Timor, Asia	Case report	1	1	100	7 years	Fever, headache, fatigue, anorexia.	Blood smear, RDT, ELISA (IgM)	*Plasmodium falciparum* and DENV (no serotype)
4	[22]	Tanzania, Africa	Analytical cross-sectional studies	364	31	08.5	Ages between 2 and 13 years	Fever, measured axillary or rectal temperature (37.5 or 38°C / 99.5 or 100.4°F)	Blood smear, ELISA (IgM/IgG), PCR	*Plasmodium* sp., and DENV (no serotype)
5	[23]	Thailand, Asia	Case report	1	1	100	11 years	Fever, chills.	Blood smear, ELISA (NS1/ IgM/IgG)	*Plasmodium falciparum* and DENV (no serotype)
6	[24]	Malaysia, Asia	Case report	1	1	100	59 years	Fever, headache, myalgia, arthralgia, and poor oral intake.	Blood smear, ELISA (NS1/ IgM)	*Plasmodium knowlesi* and DENV (no serotype)
7	[26]	Nigeria, Arica	Analytical cross-sectional studies	60	1	1.67	Ages between 3 and 70 years	Fever, chills, headache, joint, muscle and body pains.	Blood smear, RDT, PCR, ELISA (NS1/ IgM/ IgG)	*Plasmodium vivax* and *P. falciparum*, DENV (no serotype)
8	[28]	Pakistan, Asia	Analytical cross-sectional studies	856	17	1.99	Ages between 12 and 32 years	Fever lasting 2-10 days, myalgia, arthralgia, retro-orbital pain.	Blood smear, RT-PCR, ELISA (NS1/ IgM)	*Plasmodium vivax* and *P. falciparum* and DENV-2
9	[29]	India, Asia	Analytical cross-sectional studies	223	9	4.03	Ages between 22 and 56 years	Acute febrile illness (< 2 weeks), associated with nausea, vomiting, and headache.	Blood smear, ELISA (IgM)	*Plasmodium vivax* and *P. falciparum*, DENV (no serotype)
10	[30]	India, Asia	Case report	1	1	100	42 years	Fever, chills, rigor, profound generalized weakness for the last 3 days, altered sensorium (cerebral malaria and dengue).	Blood smear, RDT, ELISA (NS1 IgM/ IgG)	*Plasmodium falciparum* and DENV (no serotype)
11	[31]	India, Asia	Case report	2	2	100	35 and 63 years	Fever, chills, and stiffness for the last four days, with vomiting and abdominal pain.	Blood smear, ELISA (NS1/IgM/IgG)	*Plasmodium vivax* and DENV-2, DENV-3
12	[32]	Nigeria, Africa	Analytical cross-sectional studies	340	2	0.59	All ages	Febrile complaints (temperature > 37.5°C / 99.5°F).	Blood Smear, ELISA (IgM)	*Plasmodium* sp., and DENV (no serotype)
13	[34]	Pakistan, Asia	Analytical cross-sectional studies	159	5	3.14	Ages > 12 years	Acute febrile illness and thrombocytopenia.	Blood smear, IgM	*Plasmodium falciparum* and DENV (no serotype)

CC BY

Num.	Citation	Place/Continent	Study design	N	Positive for coinfection	Coinfection (%)	Demography	Symptomatology	Diagnostic test ML/ DENV	Remarks
14	[35]	Yemen, Asia	Analytical cross-sectional studies	270	82	30.37	Ages between 15 and 60 years	Episodes of fever, headache, arthralgia, myalgia, and retro-orbital pain.	Blood smear, RDT, ELISA (IgM/IgG)	*Plasmodium falciparum* and *P. vivax,* DENV (no serotype)
15	[36]	Brazil, South America	Analytical cross-sectional studies	72	30	41.67	Ages between 20 and 44 years	Acute febrile syndrome.	Blood smear, PCR/ RT-PCR, ELISA (IgM/ NS1)	*Plasmodium vivax*, *P. falciparum* and DENV-1, DENV-2, DENV-3, DENV-4
16	[37]	Brazil, South America	Analytical cross-sectional studies	1,578	44	2.79	Ages between < 14 and 60 years	Episodes of fever in the past 10 days.	Blood smear, RT-PCR,ELISA (IgM/NS1)	*Plasmodium vivax* and DENV-2 , DENV-4
17	[38]	Pakistan, Asia	Analytical cross-sectional studies	213	24	11.27	Ages between 13 and 70 years	Fever ≤ 10 days duration, severe body aches, rash and bleeding.	Blood smear, ELISA (IgM/IgG)	*Plasmodium vivax* and *P. falciparum*, DENV (no serotype)
18	[39]	French Guiana, South America	Analytical cross-sectional studies	208	104	50.00	Ages between 15 and 75 years	Episodes of fever above 40°C, tachycardia, initial hypotension, nausea.	Blood smear, RT-PCR, ELISA (NS1/ IgM/IgA)	*Plasmodium vivax*, *P. falciparum*, and DENV-1, DENV- 2/DENV-3
19	[40]	Bangladesh, Asian	Analytical cross-sectional studies	720	1	0.14	4 years	Febrile patients >38°C, headache, body aches, muscle pain.	RDT compatible with blood smear, ELISA (IgM)	*Plasmodium vivax* and DENV (no serotype)
20	[36]	Brazil, South America	Analytical cross-sectional studies	132	11	8.33	Ages between 16 and 92 years	Febrile, chills, myalgias, arthralgias, headache.	Blood smear, RT-PCR, ELISA (NS1)	*Plasmodium vivax* and DENV-3, DENV-4
21	[42]	Malaysia), Asia	Case report	1	1	100	59 years	Dyspnoea, chest discomfort, dry cough.	Blood smear, PCR, ELISA (NS1)	*Plasmodium knowlesi* and DENV (no serotype)
22	[43]	India, Asian	Case report	1	1	100	27 years	Myalgia (1 day before returning home to California from India after a 3 month stay in that country).	Blood smear, ELISA (IgM/ IgG)	*Plasmodium vivax* and DENV (no serotype)
23	[44]	India, Asia	Analytical cross-sectional studies	2,547	11	0.43	≥ 18 years	Febrile illness with duration of 5-14 days, rash, hepatomegaly and abdominal pain.	Blood smear, RDT, ELISA (NS1/ IgM)	*Plasmodium* sp., and DENV (no serotype)
24	[45]	Peru, South America	Analytical cross-sectional studies	95	17	17.89	Ages between 5 and 17 years	Fever measured axillary > 37.5°C, abdominal pain, nausea, vomiting.	Blood Smear, PCR, ELISA (IgM/IgG) immunofluorescence	*Plasmodium vivax*, *P. falciparum and* DENV-1, DENV-3
25	[46]	India, Asia	Case report	1	1	100	26 years	Fever, headache, severe body pain and nausea for 10 days, chills every other day.	Blood smear, ELISA(IgM/IgG)	*Plasmodium vivax* , *P. falciparum* and DENV (no serotype)
26	[47]	India, Asia	Case report	1	1	100	17 years	Fever, chills in the last 5-6 days, abdominal pain, and vomiting.	Rapid micro-agglutination test (RMAT), PCR, ELISA (IgM)	*Plasmodium vivax*, *P. falciparum* and DENV (no serotype)

Num.	Citation	Place/Continent	Study design	N	Positive for coinfection	Coinfection (%)	Demography	Symptomatology	Diagnostic test ML/ DENV	Remarks
27	(48)	Brazil, South America	Analytical cross-sectional studies	111	2	1.80	Ages > 18 years	Episodes of fever, headache, and shivering.	Blood smear, RT-PCR, Nested-PCR	*Plasmodium vivax*, *P. falciparum* and DENV-1
28	(49)	Brazil, South America	Case report	1	1	100	52 years	Chills, fever, headache, arthralgia, myalgia, choluria.	Blood smear, RDT, PCR, ELISA (IgM/ IgG/NS1)	*Plasmodium ovale* and DENV (no serotype)
29	(50)	Thailand, Asia (Burmese border)	Analytical cross-sectional studies	209	1	0,48	Ages between 15 and 41 years	Febrile episodes up to 3 days (aural temperature 37.5ºC), headache, anorexia, muscle pain.	Blood smear, ELISA (IgM/ NS1)	*Plasmodium falciparum*, *P. vivax* and DENV (no serotype)
30	(51)	Indonesia, Asia	Case report	1	1	100	49 years	Fever, chills, rigors, myalgia.	Blood smear, ELISA (IgM/ NS1)	*Plasmodium falciparum* and DENV (no serotype)
31	(52)	Ghana, Africa	Analytical cross-sectional studies	218	7	3.21	Ages between 2 and 14 years	Febrile illness, headache, nausea, chills.	RDT, ELISA (IgM/ IgG), RT-PCR	*Plasmodium* sp., and DENV (no serotype)
32	(53)	India, Asia	Case report	3	3	100	Ages between 8 months and 12 years	Fever for 5-8 days, cough, and bodyache.	Blood smear, RDT ELISA (NS1, IgM/IgG)	*Plasmodium vivax* and DENV (no serotype)
33	(54)	India, Asia	Case report	1	1	100	25 years	Fever, chills, myalgias, headache, severe headache, and high fever of 102°F (38.9°C).	Malaria Ag (pLDH/ HRP2), blood smear, ELISA (IgM)	*Plasmodium vivax*, *P. falciparum* and DENV (no serotype)
34	(59)	Spain, Europe	Case report	1	1	100	27 years	Fever, constipation, and joint pain.	Blood smear, RT-PCR, ELISA (IgM/ IgG/NS1)	*Plasmodium falciparum* and DENV-4
35	(55)	Cambodia, Asia	Analytical cross-sectional studies	9,997	15	0.15	Ages between 8 and 17 years	Fever in the last 24 hours and for < 10 days, muscle pain.	Blood smear, ELISA (IgM/IgG), Nested PCR,	*Plasmodium falciparum*, *P. vivax* and DENV-1, DENV-2, DENV-3, DENV-4
36	(56)	Nigeria, Africa	Analytical cross-sectional studies	188	19	10.11	Ages between 4 and 82 years	Acute fever (> 38ºC)	NM ELISA (IgG/IgM,NS1)	*Plasmodium* sp., and DENV (no serotype)
37	(57)	French Guiana, South American	Analytical cross-sectional studies	1723	17	0.99	NM	Episodes of fever lasting up to 4 days.	Blood smear, ELISA (IgM), RT-PCR, virus isolation	*Plasmodium vivax*, *P. falciparum and P. malariae*, DENV-1, DENV-3
38	(58)	France, Europe	Case report	1	1	100	37 years	Fever, conjunctival jaundice, vomiting, diarrhoea.	Blood smear, IgM/IgG ELISA	*Plasmodium falciparum* and DENV-3
39	(60)	Nigeria, Africa	Analytical cross-sectional studies	176	5	2,84	Ages between < 10 and 70 years	Febrile illness	RDT, ELISA(IgM ELISA) RT-PCR	*Plasmodium* sp., and DENV (no serotype)
40	(61)	India, Asia	Analytical cross-sectional studies	469	27	5.76	NM	Fever for < 7 days, running nose, myalgia, headache and bleeding manifestations.	Blood smear, ELISA (IgM/ NS1)	*Plasmodium falciparum*, *P. vivax* and DENV (no serotype)

Num.	Citation	Place/Continent	Study design	N	Positive for coinfection	Coinfection (%)	Demography	Symptomatology	Diagnostic test ML/ DENV	Remarks
41	(62)	India, Asia	Analytical cross-sectional studies	1,564	58	3.71	≥ 5 years	Fever temperature ≥ 38°C (100.4°F) and febrile illness of 2–14 days duration.	Blood smear, ELISA (IgM/IgG/NS1) and blood cultures.	*Plasmodium falciparum* and DENV (no serotype)
42	(63)	Cambodia, Asia	Analytical cross-sectional studies	1,193	27	2.26	Ages between 7 and 49 years	Febrile illness (> 38ºC), sore throat, cough, and running nose.	RDT, Nested-PCR and RT-PCR.	*Plasmodium vivax*, *P. falciparum* and DENV (no serotype)
43	(66)	Cameroon, Africa	Analytical cross-sectional studies	349	68	19.48	Ages between 6 months to 15 years	Children presenting episodes of fever (37.8 - 41°C / 100.04 - 105.8°F), vomiting, diarrhoea, and fatigue.	Blood smear, ELISA (NS1/ IgM/IgG)	*Plasmodium falciparum*, *P. vivax* and DENV (no serotype)
44	(67)	Nigeria, Africa	Analytical cross-sectional studies	529	35	6.62	Ages between < 18 and 58 years	Episodes of fever (axillary temperature > 37.8°C / 100.04°F).	Blood smear, ELISA (IgM/ IgG/ NS1)	*Plasmodium falciparum* and DENV (no serotype)
45	(75)	India, Asia	Analytical cross-sectional studies	100	3	3.00	Ages between 5 and ≥ 60 years	Fever, abdominal pain, and bleeding.	Blood smear, RDT/ ELISA	*Plasmodium* sp., and DENV (no serotype)
46	(69)	India, Asia	Analytical cross-sectional studies	1,980	22	1.11	Ages between 5 > 15 years	Febrile illness (38.3–39.4°C / 100.94-102.92°F), headache, retro-orbital pain. Fever for 2–15 days.	Blood smear, RDT, ELISA (IgM/ IgG, NS1) RT-PCR	*Plasmodium vivax*, *P. falciparum* and DENV (no serotype)
47	(70)	India, Asia	Analytical cross-sectional studies	8,364	27	0.32	NM	Fever compatible with malaria and/or dengue.	Blood smear, ELISA (NS1/ IgM)	*Plasmodium falciparum* and DENV (no serotype)
48	(71)	India, Asia	Analytical cross-sectional studies	1141	9	0.79	Ages between 12 and 80 years	Acute febrile illness.	Blood smear, ELISA (IgM, NS1)	*Plasmodium* sp., and DENV (no serotype)
49	(72)	Bangladesh, Asia	Analytical cross-sectional studies	659	5	0.76	Ages between 0 and 90 years	Fever > 37.5°C, fatigue, dizziness, and headache.	RDT, PCR, blood smear, ELISA (IgM)	*Plasmodium falciparum*, *P. vivax*, *P. malariae* and DENV (no serotype)
50	(73)	India, Asia	Case report	1	1	100	22 years	Fever > 39°, chills, rigors, cough up to 3– days.	Blood smear, ELIS A(IgM)	*Plasmodium vivax* and DENV (no serotype)
51	(74)	India, Asia	Case report	1	1	100	28 years	Fever, chills in the last 7 days, abdominal pain, vomiting.	Blood smear, ELISA (IgM)	*Plasmodium falciparum* and DENV (no serotype)

Num: number article; N: sample size; ML/DENV: Malaria/Dengue virus coinfection; ELISA: enzyme-linked immunosorbent assay; NS1: dengue non-structural protein-1; PCR: polymerase chain reaction; RDT: rapid diagnostic test; NM: not mentioned.

TABLE II

Studies for the systematic review on the worldwide distribution, symptomatology and diagnosis of coinfections between *Plasmodium* and Chikungunya virus (CHIKV)

Num.	Citation	Place/continent	Study design	N	Positive for coinfection	Coinfection (%)	Demography	Symptomatology	Diagnostic test ML/CHIKV	Remarks
1	(6)	Senegal, Africa.	Analytical cross-sectional studies	1,3845	3	0.021	All ages between 1 and 90 years	Acute febrile illnesses (> 38 °C), headache, myalgia, eye pain, arthralgia.	Blood smear, RDT, ELISA (IgM), RT-PCR	*Plasmodium* sp. and CHIKV (no serotype)
2	(22)	Tanzania, Africa	Analytical cross-sectional studies	364	2	0.55	Ages between 2 and 13 years	Fever, measured axillary or rectal temperature (37.5 or 38°C / 99.5 or 100.4°F)	Blood smear, ELISA (IgM/ IgG), PCR	*Plasmodium* sp. and CHIKV (no serotype)
3	(26)	Nigeria, Africa	Analytical cross-sectional studies	60	9	15.00	Ages between 3 and 70 years	Fever, chills, headache, joint, muscle and body pains.	Blood smear, RDT, ELISA (IgM)	*Plasmodium vivax, P. falciparum* and CHIKV (no serotype)
4	(27)	Tanzania, Africa	Analytical cross-sectional studies	400	8	2.00	Ages between < 10 and 50 years	Fever > 38°C, headache, skin rashes, joint pain.	Blood smear, RDT, ELISA (IgM/ IgG)	*Plasmodium* sp. and CHIKV (no serotype)
5	(64)	Mozambique Africa	Analytical cross-sectional studies	163	2	1.23	Ages between 5 and > 40 years	Acute febrile illness, fever > 37.5°C, headache, arthralgia, myalgia.	RDT, ELISA (IgM/ IgG/ NS1), RT-qPCR	*Plasmodium falciparum* and CHIKV (no serotype)
6	(76)	Tanzania, Africa	Analytical cross-sectional studies	448	13	2.90	Ages between 2 and 70 years	Participants without complaints, randomly selected.	Blood Smear, ELISA (IgM)	*Plasmodium* sp. and CHIKV (no serotype)

Num: number article; N: sample size; ML/CHIKV: Malaria/Chikungunya virus coinfection; ELISA: enzyme-linked immunosorbent assay; PCR: polymerase chain reaction; RDT: rapid diagnostic test; CHIKV: Chikungunya virus.

TABLE III

Studies for the systematic review on the worldwide distribution, symptomatology and diagnosis of coinfections between *Plasmodium* and Zika virus (ZIKV), *Plasmodium* and Yellow fever virus (YFV)

Num.	Citation	Place/ Continent	Study design	N	Positive for coinfection	Coinfection (%)	Demography	Symptomatology	Diagnostic test ML/ ZIKV or ML/YFV	Remarks
1	(6)	Senegal, Africa	Analytical cross-sectional studies	1,3845	8	0.13	All ages between 1 and 90 years	Acute febrile illnesses (> 38°C), headache, myalgia, eye pain, arthralgia.	Blood smear, RDT, ELISA (IgM), RT-PCR	*Plasmodium* sp., ZIKV, DENV (no serotype), *Plasmodium* sp. and YFV
2	(25)	Nigeria, Africa	Analytical cross-sectional studies	118	15	12.71	All ages between 1 and 78 years	Fever > 37.5°C.	RDT, ELISA (IgM/IgG) Immunochromatography	*Plasmodium* sp. and ZIKV
3	(33)	Madagascar, Africa	Analytical cross-sectional studies	1,216	2	0.16	Pregnant women, all ages	NM.	ELISA (IgG/IgM) IIFA, PCR	*Plasmodium falciparum* and ZIKV

Num: number article; N: sample size; ML/ZIKV: Malaria/Zika virus coinfection; ML/YFV: Malaria/Yellow fever virus coinfection; ELISA: enzyme-linked immunosorbent assay; PCR: polymerase chain reaction; RDT: rapid diagnostic test; CHIKV: Zika virus; NM: not mentioned.

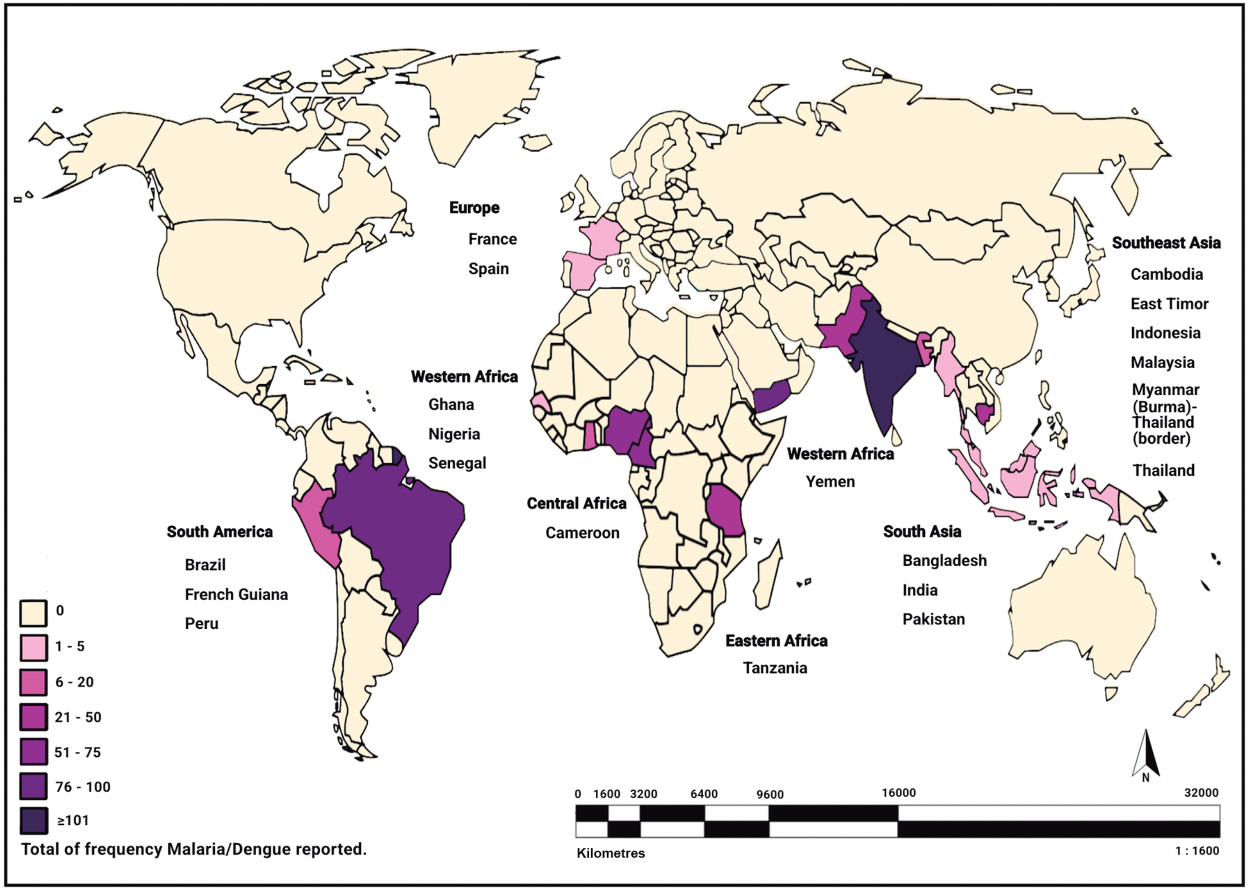

Fig. 1: worldwide frequency and distribution map of malaria (ML) and dengue (DEN) coinfections, according to the studies in this systematic review.

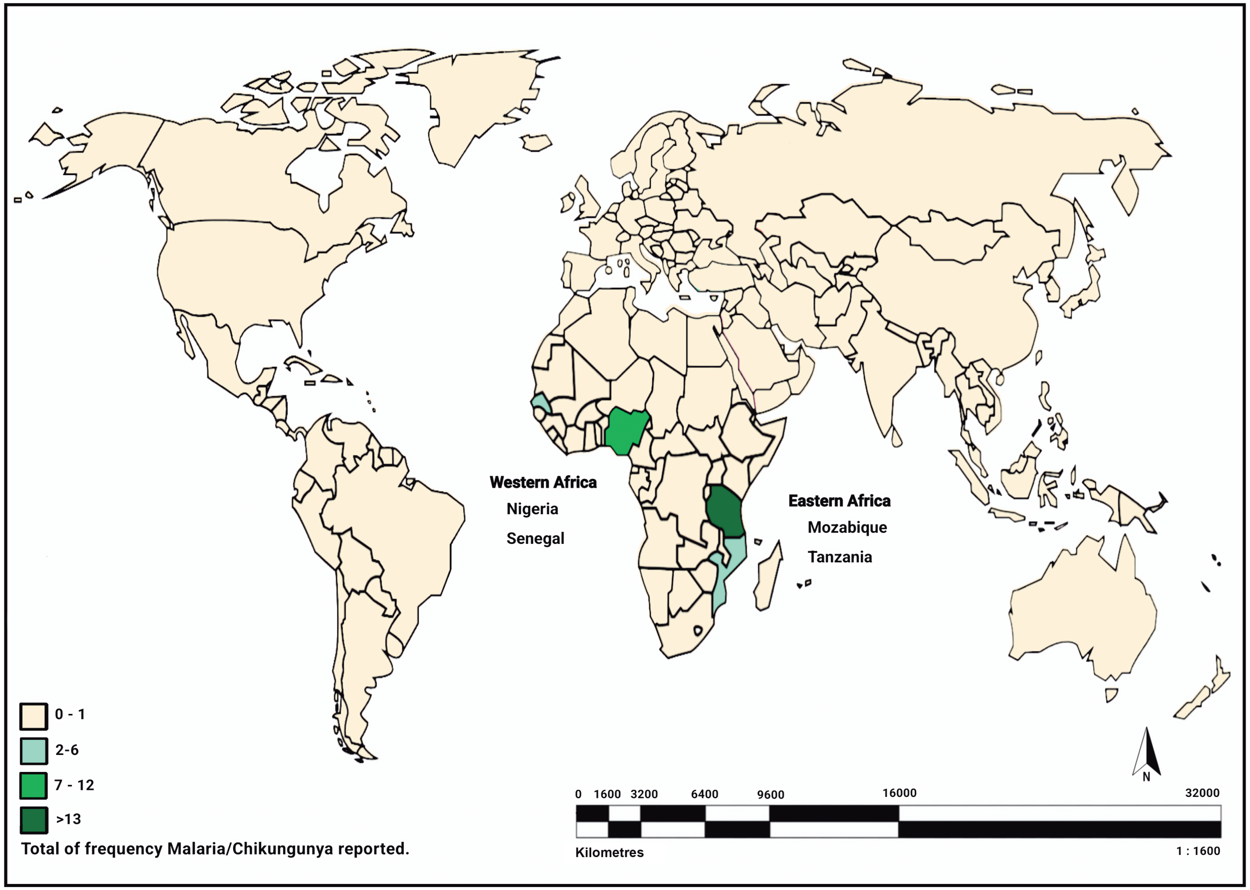

Fig. 2: worldwide frequency and distribution map of malaria (ML) and Chikungunya (CHIK) coinfections, according to the studies in this systematic review.

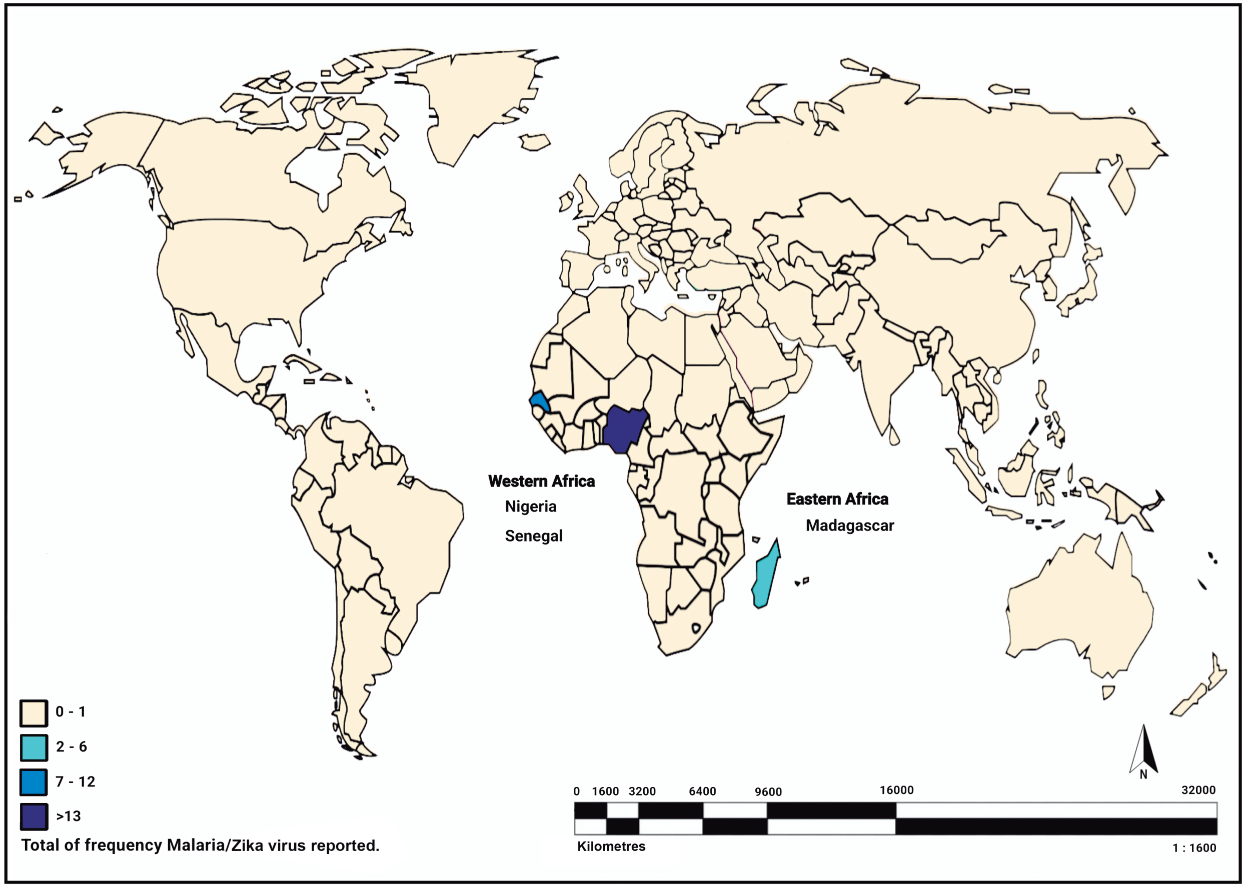

Fig. 3: worldwide frequency and distribution map of malaria (ML) and Zika (ZIK) coinfections, according to the studies in this systematic review.

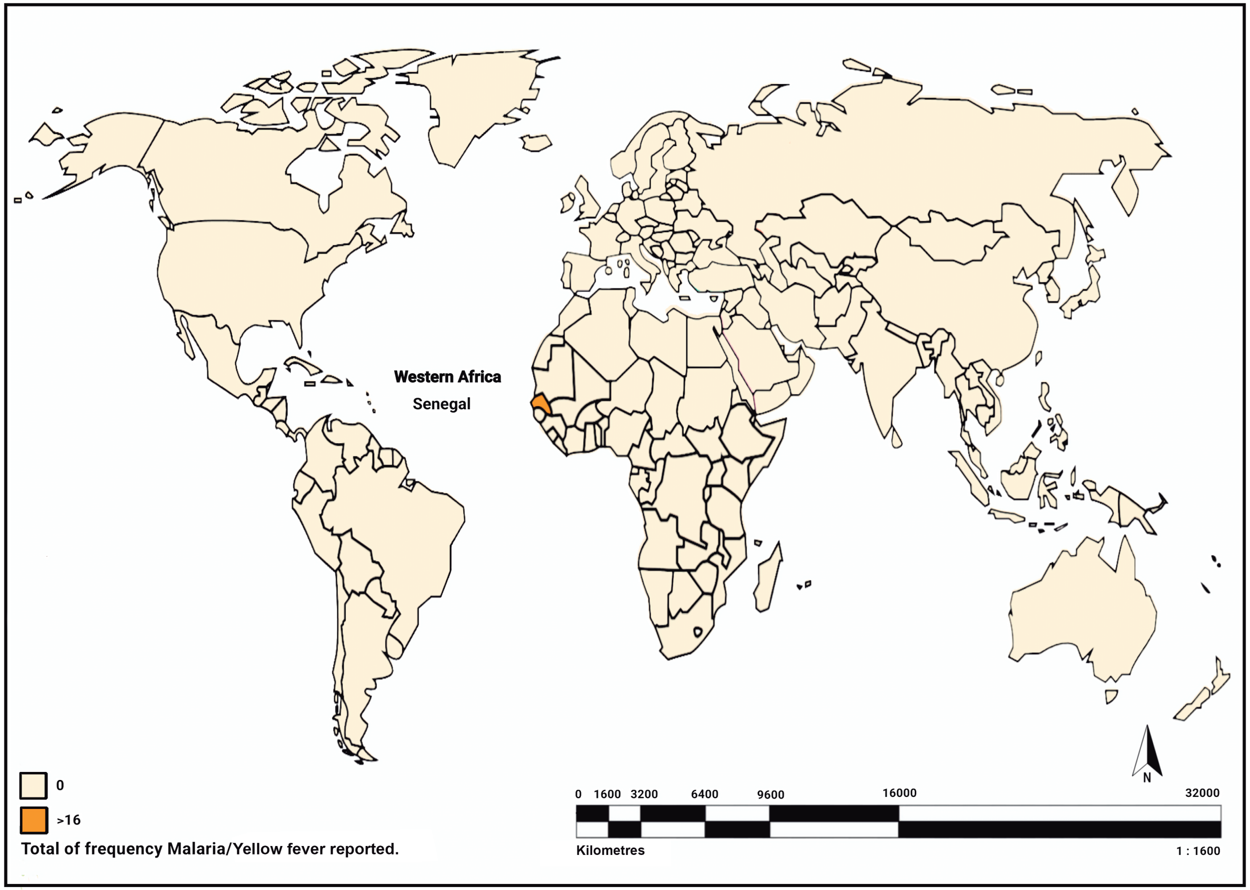

Fig. 4: worldwide frequency and distribution map of malaria (ML) and Yellow fever (YF) coinfections, according to the studies in this systematic review.
